# Editorial to “Atrial standstill in a young patient with ischemic stroke associated with inheritance of a novel HCN4 mutation”

**DOI:** 10.1002/joa3.13164

**Published:** 2024-10-07

**Authors:** Hidekazu Kondo, Naohiko Takahashi

**Affiliations:** ^1^ Department of Cardiology and Clinical Examination, Faculty of Medicine Oita University Yufu Oita Japan

Abhinav et al. reported a young patient with atrial standstill who was found to have an HCN4 genetic variant.^1^ This is the first report of an association between HCN4 genetic variant and atrial mechanical and electrical standstill, which is very significant. Several causative genes for familial progressive cardiac conduction disease (PCCD) have been reported, including the gene they reported. As they mentioned in the discussion, the SCN5A mutation is the most well‐known PCCD‐causing gene. LMNA, MYH6, and TRPM4 have also been reported as PCCD‐causing genes in addition to SCN5A.^2^, ^3^, ^4^ Furthermore, Ishikawa et al. reported that sick sinus syndrome patients with HCN4 genetic variant frequently present with atrial fibrillation (AF) and left ventricular noncompaction (LVNC), which means that genetic variant of HCN4 could be a cause of PCCD; the mechanism by which HCN4 genetic variant generate such atrial and ventricular structural and electrical abnormality is still unclear. Normally, HCN4 is expressed primarily in limited regions of the cardiac conduction system, particularly in the sinoatrial node. It plays a crucial role in slow diastolic depolarization involved in automaticity. However, during the course of early embryonic development, HCN4‐positive cells also have been shown to give rise to primitive heart tubes, which eventually form most of the myocytes in the left ventricle and part of the atrium of the adult heart. Ishikawa et al. indicated that the genetic variant in HCN4 may disrupt the normal differentiation of progenitor cells in the primitive heart, resulting in disturbance of structural and electrical properties of the left ventricle and both atria, which may ultimately be a substrate for the development of AF and LVNC.^5^ This speculation could explain how the HCN4 genetic abnormality in this case could cause structural and electrical atrial dysfunction, resulting in the phenotype of atrial standstill.

We were worried about the penetrance of this unique genetic variant. Multidisciplinary genetic counselling needs to be performed in order to perform genetic testing of his sister, parents, and aunt at an appropriate time, which could assist with the performance of appropriate intervention. Particularly, if the author has the opportunity to see his sister, we will encourage the authors to recommend that she should take a genetic testing along with electrocardiogram, which may help her prevent the development of new stroke at a young age.

If the patient has the symptoms because of chronotropic incompetence, the indication for a cardiac implantable electrical device (CIED) should be considered. However, decisions on which CIED to be implanted should be made carefully. The present case developed the atrial standstill, which limits the benefit of a dual chamber pacemaker. Since the disadvantages of ventricular lead implantation at a young age have also been proposed in recent years, we think that a leadless pacemaker might be an option for young patients with preserved left ventricular systolic function. The recently launched leadless pacemaker, AveirTM (Abbott), is retrievable and has the potential to be useful in pacemaker implantation in younger patients. However, we should take into accounts the possibility of left ventricular systolic dysfunction because of increased right ventricular pacing in addition to the patient's genetic background. Transvenous conduction system pacing or cardiac resynchronization therapy by biventricular pacing may need to be considered at the appropriate time with close follow‐up. Hopefully, more cases of HCN4 genetic variant such as the present case will be accumulated, and better treatment strategies will be defined in the future guidelines.

## CONFLICT OF INTEREST STATEMENT

Authors declare no conflict of interests for this article.
